# Host-specific symbioses and the microbial prey of a pelagic tunicate (*Pyrosoma atlanticum*)

**DOI:** 10.1038/s43705-021-00007-1

**Published:** 2021-04-14

**Authors:** Anne W. Thompson, Anna C. Ward, Carey P. Sweeney, Kelly R. Sutherland

**Affiliations:** 1 Department of Biology, Portland State University, Portland, OR USA; 2 Oregon Institute of Marine Biology, University of Oregon, Eugene, OR USA

**Keywords:** Microbial ecology, Microbial biooceanography, Microbiome, Symbiosis

## Abstract

Pyrosomes are widely distributed pelagic tunicates that have the potential to reshape marine food webs when they bloom. However, their grazing preferences and interactions with the background microbial community are poorly understood. This is the first study of the marine microorganisms associated with pyrosomes undertaken to improve the understanding of pyrosome biology, the impact of pyrosome blooms on marine microbial systems, and microbial symbioses with marine animals. The diversity, relative abundance, and taxonomy of pyrosome-associated microorganisms were compared to seawater during a *Pyrosoma atlanticum* bloom in the Northern California Current System using high-throughput sequencing of the 16S rRNA gene, microscopy, and flow cytometry. We found that pyrosomes harbor a microbiome distinct from the surrounding seawater, which was dominated by a few novel taxa. In addition to the dominant taxa, numerous more rare pyrosome-specific microbial taxa were recovered. Multiple bioluminescent taxa were present in pyrosomes, which may be a source of the iconic pyrosome luminescence. We also discovered free-living marine microorganisms in association with pyrosomes, suggesting that pyrosome feeding impacts all microbial size classes but preferentially removes larger eukaryotic taxa. This study demonstrates that microbial symbionts and microbial prey are central to pyrosome biology. In addition to pyrosome impacts on higher trophic level marine food webs, the work suggests that pyrosomes also alter marine food webs at the microbial level through feeding and seeding of the marine microbial communities with their symbionts. Future efforts to predict pyrosome blooms, and account for their ecosystem impacts, should consider pyrosome interactions with marine microbial communities.

## Introduction

Pyrosomes are globally abundant pelagic tunicates that can alter marine ecosystems, especially when they bloom. Pyrosomes graze efficiently^1–5^ and, in turn, become prey to fish,^6,7^ sea turtles,^8^ seabirds,^9,10^ and marine mammals,^11^ and provide habitats to invertebrates.^8,12^ Through sinking after death, vertical migration, and fecal pellets, pyrosomes contribute to detrital food webs, transport carbon below the mixed layer,^3^ and feed benthic megafauna.^13,14^ Thus, pyrosomes have the capacity to dramatically alter energy and carbon cycles in marine ecosystems, however, knowledge of pyrosome biology is limited.

Addressing pyrosome interactions with marine microorganisms could improve the understanding of pyrosome biology, bloom dynamics, and impacts to ecosystems because marine microorganisms are major players in animal biology,^15^ marine trophic structure,^16^ and biogeochemical cycling.^17^ As filter feeders, pyrosomes ingest numerous microorganisms. Retention of large eukaryotic phytoplankton has been established,^2,4,5,18^ but feeding on smaller abundant heterotrophic marine microbes has not been directly tested.^19^ Pyrosome bioluminescence suggests a host-specific relationship with microbial symbionts, though none have been identified.^20–22^ The microbiomes of other tunicates provide their host with nitrogen,^23^ carbon,^24^ secondary metabolites,^25^ extending the host range into nutrient poor environments. For non-tunicate gelatinous plankton, such as jellyfish and ctenophores, microbial associates can be vectors for fish pathogens.^26–28^ In addition, marine animals are a source of microbial diversity to marine ecosystems, especially for the seawater rare biosphere.^29^ These characteristics of pyrosomes and other gelatinous animals point to great potential for pyrosome–microbe interactions to influence pyrosome biology and seawater microbial communities.

Here, we examined the microbial community of pyrosomes (i.e., the pyrosome microbiome) to expand the understanding of pyrosome biology and impacts on marine microbial ecosystems. *Pyrosoma atlanticum* colonies and surrounding seawater were sampled during bloom conditions in the Northern California Current System (NCC). We measured microbial community structure and diversity via 16S rRNA gene sequencing, flow cytometry, and microscopy. The goals of this study were to: (1) compare the pyrosome microbial community to surrounding seawater, (2) investigate the roles of specific microbial taxa in pyrosome ecology, and (3) identify potential roles of pyrosomes in controlling populations of marine microorganisms through feeding. Together, these data demonstrate that pyrosomes shape, and are shaped by, marine microbial communities. Future work to account for their role in marine ecosystems, especially during blooms, should consider pyrosome–microbe interactions.

## Method and materials

### Northern California Current (NCC) System during the 2018 pyrosome bloom

Pyrosomes and seawater were collected from the NCC, off the Oregon Coast, in July 2018, near the peak of a multiyear bloom of *P. atlanticum.*^30–32^ Samples were collected from the R/V Sally Ride (SR1810) along the Newport Hydrographic Line. Station D5 (Cast 20, 44.652141N; 125.117573W) was sampled on July 9, 2018 in the presence of a strong temperature gradient in the top 20 meters and a chlorophyll peak at 17 meters (Supplementary Fig. 1A). Station D3 (Cast 26, 44.651756N; 124.589313W) was sampled on July 11, 2018 in the presence of a mixed layer depth of 15 meters and a chlorophyll peak at the base of the mixed layer (Supplementary Fig. 1B).

### Pyrosome and seawater sampling

Pyrosomes were sampled using a coupled multiple opening and closing net and environmental sensing system^33^ with mesh sizes of either 333 µm or 1 mm. Seawater was collected using a CTD Rosette with 24 12 L Niskin bottles. At Station D5, three pyrosomes were sampled between 50 and 75 meters, and seawater was collected from 90, 25, 15, and 4 meters. At Station D3, four pyrosomes were sampled between 0 and 100 meters, and seawater was collected from 90, 25, 17, and 10 meters. 1 L seawater samples were filtered through a 1.6 µm Whatman GF/A microfiber filter and collected on 0.2 µm Pall Supor PES membranes. Pyrosomes were rinsed three times with 0.2 µm filtered seawater to remove unattached microbes. Zooids were extruded from the tunic by applying digital pressure along the interior cavity of the pyrosome. The collected material (zooids, pyrosome fecal material, and mucus) was transferred to a sterile tube and allowed to settle for five min then rinsed three times with 0.2 µm filtered seawater to remove unattached microbes. Samples for flow cytometry were fixed with 0.125% TEM-grade glutaraldehyde (Tousimis) and incubated for 10 min in the dark at room temperature before freezing in liquid nitrogen. Samples for microscopy were rinsed with freshwater to prevent salt crystals from inhibiting imaging and then fixed with 0.125% TEM-grade glutaraldehyde. All samples including pyrosome tissue and seawater were archived at −20 or −80 °C.

### DNA extractions and PCR with universal 16S rRNA gene primers

DNA extraction was done using the DNeasy Plant Tissue Mini Kit (Qiagen) with the following modifications. Pyrosome tissue was ground with a sterile pestle (Axygen, Tewksbury, USA) for 3 min prior to extraction. Seawater and pyrosome samples were lysed by bead beating with 0.55 and 0.25 mm sterile glass beads at 30 Hz for 2 min after addition of lysis buffer, freeze-fractured three times, incubated with Proteinase K (VWR Chemicals, Solon, OH, USA) at 20 mg/mL for 1 h at 55 °C, and incubated with RNase A at 100 mg/mL for 10 min at 65 °C. To minimize amplification of eukaryotic host DNA, the primer pair 515F‐Y/806R was chosen to amplify the 16S rRNA V4 hypervariable region with conditions as published.^34^ Reactions were performed with 0.5–2 ng of DNA using the QuantaBio 5Prime HotMasterMix (Qiagen Beverly, MA, USA). The Agilent High Sensitivity Kit in the Bioanalyzer (Agilent Technologies, Waldbronn, Germany) confirmed amplicon size. Triplicate reactions from each sample were pooled and paired-end sequenced with Illumina MiSeq v.3 (Illumina, San Diego, USA).

### Identification of amplicon sequence variants (ASVs)

**Fig. 1:**
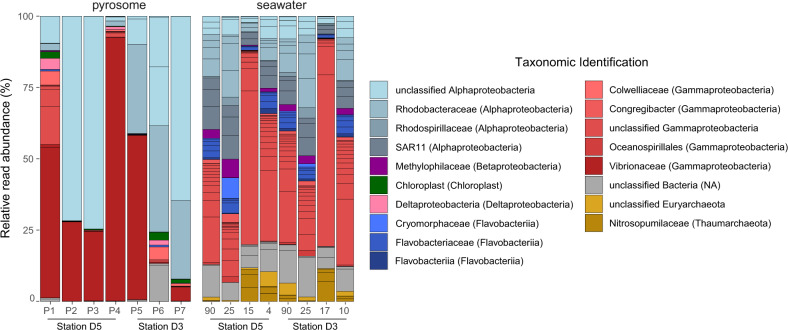
Top 50 most abundant ASVs in pyrosome and seawater samples colored by class and genus. Individual ASVs within the same genus are indicated by horizontal black lines. Individual pyrosome samples are designated by the station where they were sampled and a unique identifier (i.e., P1 = pyrosome 1). Individual seawater samples are designated by the station where they were sampled and the depth of collection in meters.

The program *dada2*^35^ was used to identify unique ASVs from raw sequence reads. Using the function *filterAndTrim*, forward and reverse primer sequences were removed, maxEE was set to 2, trunQ was set to 11, maxN was set to 0. The function *orient.fwd* was used to orient sequences in the same direction. About 30% of sequence reads were removed from each sample due to low quality. Error rates of forward and reverse reads were modeled with *learnerrors* for 100 Million bases. Paired ends were merged with *mergePairs* and unique sequences were inferred with the function *dada*. Chimeras were removed with *removeBimeraDenovo* using the “consensus” method. Taxonomy of the ASVs to the species level was performed with the function *assignTaxonomy* against RefSeq+RDP version 2,^36–38^ with 50 as the minimum bootstrap confidence for assigning a taxonomic level. In addition to Bacteria and Archaea sequences, chloroplast sequences from eukaryotic phytoplankton were examined. The similarity of ASVs to known sequences from environmental and microbial isolates was determined through NCBI BLASTn^39^ against NT and ENV_NT. For each ASV, we retained information from the top 20 best hits including percent identity, accession number, and sample source (i.e., host, sample, metadata). The *phyloseq* package^40^ was used to combine, analyze, and graphically display ASV tables (Fig. 1). Raw sequence data were deposited to BioProject PRJNA659246. Data on ASV taxonomy, sample attributes, and quality-filtered raw read abundance are in Supplementary Tables 1 and 2 and Supplementary Figs. 2 and 3.

### Diversity metrics

**Fig. 2:**
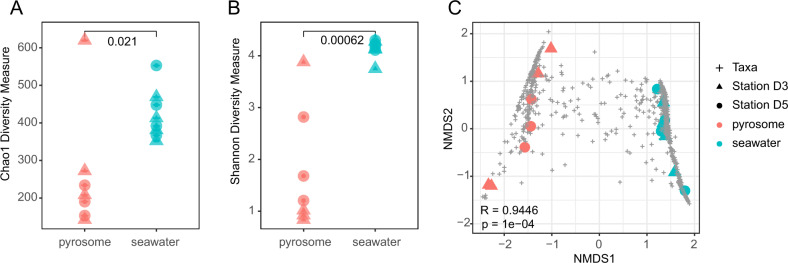
The pyrosome microbiome is less diverse and distinct from surrounding seawater. Alpha diversity metrics for Chao1 (**A**) and Shannon diversity (**B**) were significantly less for pyrosomes than surrounding seawater (*p* values < 0.05, Wilcox test). NMDS of Bray Curtis Dissimilarity (**C**) showed strong differences between seawater and pyrosomes (ANOSIM). Most ASVs (taxa) clustered with pyrosomes or seawater, and a few ASVs overlapped between the two sample types. Supplementary Fig. 4 shows similarity of pyrosome diversity across depths and significant changes in seawater diversity over depth.

Alpha and beta diversities were calculated to determine differences in microbial community structure between pyrosomes and seawater. Alpha diversity measures were performed on the non-rarefied, non-standardized, data set to allow for the influence of rare ASVs and singletons. Chao1 and Shannon measures of alpha diversity were calculated and visualized using the function *plot_richness* in *phyloseq*^40^ using the wilcoxon test for significance. Analysis of beta diversity was performed on ASV relative abundance standardized to median size of the sequence libraries. The *phyloseq* function *ordinate* was used to calculate Bray Curtis dissimilarities and visualize the results with NMDS. Analysis of similarity (ANOSIM)^41^ was used to test significance (*p* value) and strength of clustering (R statistic) by sample type and depth, which are displayed in Fig. 2 and Supplementary Fig. 4.

### Differential abundance analysis

*DESeq2*^42^ was used to identify ASVs that were significantly differentially abundant in pyrosomes compared to seawater following recommendations for microbiome data.^43^ *DESeq2* was performed on the non-rarefied data set of ASV counts standardized to the median size of the sequence libraries (Supplementary Table 3 and Supplementary Fig. 5). The significance of differences between pyrosomes and seawater for each ASV was assessed with the Wald test using the Benjamini–Hochberg (BH) adjustment for multiple tests. BH adjusted *p* values < 0.01 were called “significantly differentially abundant,” and ASVs with *p* values ≥ 0.01 were designated “not significantly differentially abundant”. Significantly differentially abundant ASVs with positive log2(fold change) were designated “pyrosome enriched” and those with negative log2(fold change) were designated as “seawater enriched”.

### Categorizing ASVs by potential ecological role

**Fig. 3:**
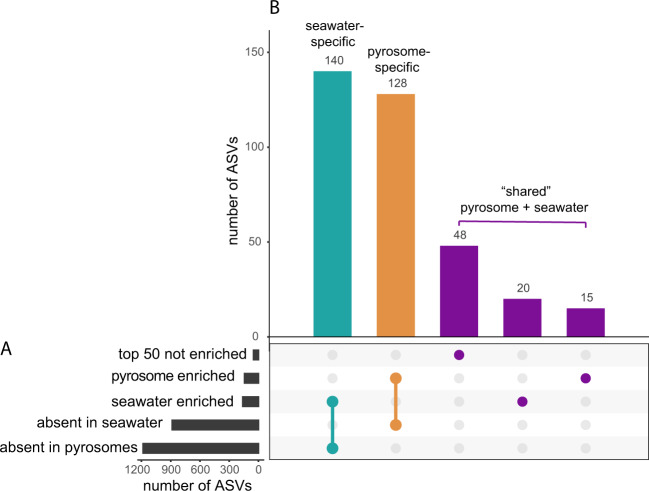
Categorization of ASVs based on their relative abundance and distribution across pyrosomes and seawater. **A** Black bar plots show the number of ASVs in each individual category from the total of 2284 ASVs detected in all samples. “Enriched” (or “not enriched”) refers to differential abundance analysis between seawater and pyrosome samples with DE*Seq*2. “Top 50” are the fifty most abundant ASVs that were not differentially abundant between the two sample types. **B** Colored dots indicate the category (or, categories), represented in the colored bar plots of defined ASV groups including “seawater-specific”, “pyrosome-specific”, or “shared” ASVs. A subset of specific ASVs from each category (bar plots, (**B**)) is discussed further in the text.

ASVs were categorized by their distribution across pyrosomes and seawater, and similarity to known sequences (Supplementary Fig. 6) to generate hypotheses on their ecological roles. The number of ASVs in each category is displayed in Fig. 3. Pyrosome-specific ASVs were considered as potential symbionts, broadly defined to include commensal, mutualistic, pathogenic, and parasitic interactions^44^ (Supplementary Table 4). “Shared ASVs” were present in both seawater and pyrosome samples, and could either be significantly differentially abundant in the two sample types or not (Supplementary Table 5). Analysis of shared ASVs was limited to the most abundant. Shared ASVs were considered potential symbionts or consumed prey. We identified ASVs that were present in all pyrosomes (100% of pyrosome samples) as the pyrosome “core” microbiome, regardless of their presence in seawater (Supplementary Table 6). This definition of core is stringent compared to other studies, such as in sponges,^45^ especially given the compositional nature (i.e., relative abundance) of our data set. Pyrosome core ASVs were considered potential symbionts or potential consumed prey. “Seawater-specific ASVs” were significantly enriched in seawater relative to pyrosomes (via *DESeq2*) and were absent from all pyrosomes (Supplementary Table 7) and were considered to belong to non-pyrosome-associated microbial taxa. We acknowledge the caveat of the compositional nature of the 16S rRNA gene data set,^46^ which requires semiquantitative or qualitative interpretation.

### Phylogeny of putative bioluminescent taxa

**Fig. 4:**
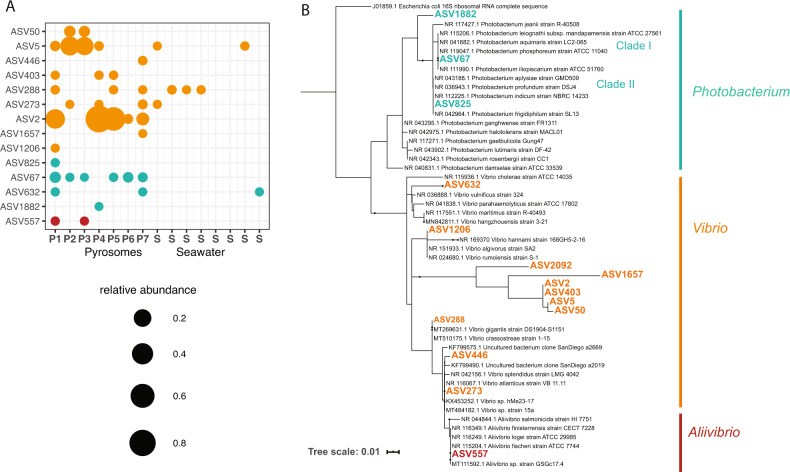
Pyrosomes contain ASVs from multiple lineages of bioluminescent taxa. **A** Percent relative abundance of possible bioluminescent ASVs across pyrosomes samples (P) and seawater samples (S). **B** Maximum likelihood tree of 16S rRNA gene of ASVs relative to reference sequences for *Vibrio*, *Photobacterium*, and *Aliivibrio*.

Phylogenetic analysis of ASVs belonging to Vibrionacae genera *Vibrio*, *Aliivibrio*, and *Photobacterium* was performed to study putative bioluminescent taxa in the pyrosome microbiome (Fig. 4 and Supplementary Table 8). Reference sequences from each genera were chosen as in previous studies^47^ using *E. coli* as an outgroup. Molecular Evolutionary Genetics Analysis (MEGA v10.1.8)^48^ was used to align with ClustalW (240 bp) and construct maximum likelihood trees with 1000 bootstraps using the Hasegawa–Kishino–Yano (1985) model. Phylogenetic trees were annotated and visualized with iTOL v5.^49^

### Flow cytometry

Flow cytometry was used to quantify the pigmented cells (i.e., phytoplankton) in the pyrosomes and seawater. Pyrosome flow cytometry samples were pulverized with a sterile pestle and filtered through a 50 µm filter to remove tissue that would clog the flow cytometer. A BD influx high-speed cell sorter (BD Biosciences, San Jose, CA) equipped with a small particle detector collected data triggered on forward-scattered light (FSC). For each particle, data on FSC, red fluorescence (chlorophyll), and orange fluorescence (phycoerythrin) were collected. Flow cytometry data were analyzed using FlowJo v10.6.2 and R v3.6.1 and deposited into *FlowRepository* #FR-FCM-Z3YE. Pigmented cells were defined based on their fluorescence signals above the autofluorescence of the macerated pyrosome tissue. Cells were classified as pigmented picoeukaryotes (PPE, chlorophyll only) or *Synechococcus* (chlorophyll and phycoerythrin). Welch’s *t* test was used to compare the relative abundance of *Synechococcus* to PPE in seawater vs. pyrosomes.

### Microscopy

Triplicate 10 µL aliquots of preserved pyrosome samples were analyzed using compound light microscopy and environmental scanning electron microscopy (eSEM). A Nikon Epifluorescence microscope equipped with a Canon EOS 5D camera was used to capture images of cells greater than 10 µm in diameter. A FEI Quanta eSEM microscope was used to capture images of cells < 10 µm. Images of whole cells were identified by morphology and size.^50–52^ Cells were identified into broad groupings: diatoms (single vs. chain, pennate vs. centric), flagellates, nanozooplankton, mixed assemblages, and unidentified. Cells captured using eSEM were also identified to the genus level where visibly distinct. Brightness and contrast of images were adjusted using ImageJ v1.52k or Adobe Illustrator v24.1.

## Results and discussion

### Pyrosome-specific microbial community

This is the first study to report on the microbial communities of the entire organism of Thaliacean Order Pyrosomidae (pyrosomes). In total, 4,437,471 high-quality sequences were obtained from pyrosomes and seawater with a median of 268,634 reads per sample (Supplementary Fig. 2A). From these sequences, 2284 unique ASVs were discovered from 25 phyla. Most sequences belonged to Proteobacteria (80%), Bacteroidetes (6%), or Bacteria unclassified at the phylum level (6%) (Supplementary Fig. 2B).

Major differences were evident between pyrosome and seawater microbial communities, suggesting pyrosome-specific associations with marine microorganisms. Pyrosomes were dominated by three ASVs from Alphaproteobacteria and Gammaproteobacteria lineages (Fig. 1A). In contrast, seawater contained numerous closely related ASVs from many seawater-associated lineages including SAR11, Flavobacteriia, Euryarchaeota, and Thaumarchaeota (Fig. 1B). Pyrosome microbial communities were significantly less diverse than seawater in richness (Chao1, *p* value = 0.02) and richness and evenness (Shannon, *p* value < 0.01) (Fig. 2A), though pyrosome microbiomes spanned a greater range of alpha diversities than seawater samples. The low diversity of pyrosomes relative to seawater was similar to observations of ctenophores^53^ and jellyfish,^54^ but contrasted with the high diversity of benthic sponge and coral microbiomes relative to seawater.^55,56^

Beta-diversity analysis revealed pyrosome-specific microbial community structure, distinct from seawater (ANOSIM R statistic = 0.95, *p* value < 0.01), which included thousands of less abundant microbial taxa in addition to the dominant taxa (Fig. 2C). Seawater microbial communities partitioned significantly by depth (*p* value < 0.01, Supplementary Fig. 4B) likely due to vertical stratification at the study site. In contrast, pyrosome microbial communities were similar between stations and depths suggesting that host properties more strongly control pyrosome microbial communities than exposure to surrounding seawater (Supplementary Fig. 4C, D).

### Two novel taxa dominate the pyrosome microbiome

Pyrosome microbial communities were numerically dominated by two ASVs matching Alphaproteobacteria (ASV1) and Gammaproteobacteria (ASV2) (Fig. 1). At the extreme, a single ASV accounted for 92% of the sequences recovered from one pyrosome (Fig. 1, pyrosome 4). ASV1 was shared between pyrosomes and seawater and was part of the pyrosome core microbiome (Supplementary Tables 5 and 6 and Supplementary Fig. 7). Classified as an Alphaproteobacterium, ASV1 was only 90% similar to existing sequences, which included symbionts from a gutless marine annelid (Siboglinidae) known to depend on high densities of microbial chemolithotrophic endosymbionts for nutrition.^57^ ASV2 was exclusively present in pyrosomes, but not part of the pyrosome core. ASV2 belonged to the Order Vibrionaceae, Genus *Vibrio*, but was only 92.1% similar to existing sequences from fish guts and corals (Supplementary Table 4). The dominance of these two ASVs suggests that numerous cells from these novel taxa associate with pyrosomes as broadly defined symbionts.^44^

Aside from these two dominant ASVs, additional abundant pyrosome-specific and shared ASVs were novel compared to existing sequence databases. Similar to ASV2, ASV5 was one of the most abundant shared ASVs, but was distinct (92.1% identity) from other known *Vibrio* (Fig. 4 and Supplementary Table 5). Pyrosome-specific ASV7 was classified as an Alphaproteobacterium with 88.8% similarity to sequences from coastal seawater, symbionts of the tunicate *Ciona intestinalis*, and symbionts of the coral *Gorgonia ventalina* (Supplementary Table 4). Similarly, pyrosome-specific and core ASV12 was 79.9% similar to existing sequences including seawater near coral reefs and the sponge *Tethya californiana* (Supplementary Table 4). These abundant pyrosome ASV sequences were distinct from known symbionts of other marine invertebrates suggesting that pyrosomes contain unique symbionts. Study of the microbiomes of other pelagic tunicates will reveal whether these ASVs are unique to pyrosomes.

An exception to the pattern of dominance by a few ASVs was an individual pyrosome where many diverse ASVs composed 50% of its sequence reads (pyrosome 1, Fig. 1). This pyrosome matched or exceeded seawater samples in alpha diversity (Fig. 2A and Supplementary Fig. 3), indicating that pyrosome associations with dominant symbionts may be dynamic, zooid, colony, or condition specific. Dynamic infection of animal hosts by symbionts or pathogens has been shown in other animal–microbe systems. In the Hawaiian bobtail squid-*Aliivibrio* symbiosis, the number of symbionts recruited from seawater determines host colonization levels.^58^ Alternatively, variation in dominant symbionts across individual pyrosomes could indicate microbiome structuring at the individual colony level, similar to observations in corals.^55^

### Pyrosome-specific symbionts and seawater-derived symbionts form the pyrosome microbiome

To generate additional hypotheses on the ecological role of the ASVs, we defined four groups of ASVs based on their distribution in pyrosomes and seawater. First, we discovered 128 “pyrosome-specific” ASVs (Fig. 3 and Supplementary Table 4), which may be symbionts that lack a free-living seawater phase. Well-represented microbial lineages in the pyrosome-specific group included Actinomycetales, Cytophagales, Flavobacteriales, Sphingobacteriales, Planctomycetes, Rhodobacterales, Myxococcales, Desulfovibrionales, Pseudomonadales, Vibrionales, and Verrucomicrobiales. These ASVs were most similar to sequences from a wide range of sources including seawater, marine, and terrestrial animals, and have been recovered from the microbiomes of hosts including corals, sponges, and sea squirts (Supplementary Fig. 6 and Supplementary Table 4). Second, we discovered 83 “shared ASVs” defined as ASVs significantly enriched in seawater and present in pyrosomes (*n* = 20), enriched in pyrosomes and present in seawater (*n* = 15), or numerous, but not significantly differentially abundant between pyrosomes and seawater (*n* = 48) (Fig. 3). We hypothesize that shared ASVs are pyrosome symbionts with a free-living seawater phase or retained microbial prey from filter feeding. Shared ASVs matched existing sequences from primarily seawater sources with some best hits to symbionts of animal hosts (Supplementary Fig. 6 and Supplementary Table 5). Microbial lineages among shared ASVs included Flavobacteriales, Planctomycetes, SAR11, Rhodobacterales, Rhodospirillales, Alteromonadales, and Nitrosopumilales (Supplementary Table 5). Next, we discovered 21 ASVs that comprised the pyrosome core microbiome and were either absent in seawater (pyrosome specific) or present in seawater (shared). Pyrosome core ASVs may be integral to pyrosome biology as symbionts or as microbial prey. Lastly, we discovered 140 “seawater-specific ASVs” defined as ASVs significantly enriched in seawater samples and absent in pyrosomes (Fig. 3). We hypothesize that seawater-specific ASVs are free-living microorganisms not consumed by pyrosomes. Seawater-specific ASVs were highly similar to existing sequences from ubiquitous marine microbial lineages (Supplementary Fig. 6 and Supplementary Table 7). Together these results demonstrate that the pyrosome microbiome is a combination of host-specific symbionts that exist without a free-living seawater phase and microbial taxa that are either recruited from seawater or grazed as prey. The interaction of pyrosomes with a complex assemblage of microorganisms reflects what is understood for benthic tunicates^23–25^ but contrasts with non-filter-feeding gelatinous animals such as Ctenophora (comb jellies) which have no, or few, symbionts.^59,60^

### Living host to a complex biofilm microbial community

Pyrosomes contained ASVs from several microbial lineages implicated in the colonization or control of marine biofilms including *Amylibacter ulvae*, Myxococcales, Actinobacteria, and Planctomycetes (Supplementary Tables 4 and 5). Specifically, ASV3 was identical to *Amylibacter ulvae*, which was isolated from a green alga^61^ and may contribute to algal morphology development, disease resistance, nutrient provision, and spore release,^62^ and is abundant in biofilms on marine surfaces.^63^ ASV3 was the third most abundant taxa in pyrosomes and was also abundant in seawater (Supplementary Fig. 7), consistent with the prevalence of *Amylibacter sp*. in diverse coastal marine ecosystems.^64^ Planctomycetes were represented in the pyrosome core and pyrosome-specific microbiota by eight ASVs from the genera *Rhodopirellula*, *Phycisphaera*, and *Blastopirellula* (Supplementary Tables 4–6). In the whole data set, Planctomycetes were represented by over 50 ASVs, most of which were exclusively present, or enriched, in pyrosomes relative to seawater (Supplementary Fig. 8). Planctomycetes colonize marine surfaces including algae,^65^ sponges,^66,67^ ascidians,^68^ and corals^56,69,70^ through resistance to several antibiotics.^71^ The numerous Planctomycete ASVs discovered in pyrosomes mirrors macroalgae microbial communities,^72^ thus Planctomycetes may control pyrosome surface-associated biofilms, participate in carbon cycling, and consume specific polysaccharides produced by pyrosomes. Another group of surface-related taxa among pyrosome-specific ASVs was of the Order Actinomycetales (Supplementary Table 4), a lineage prevalent in marine and freshwater habitats^73,74^ and common symbionts of marine invertebrates. Pyrosome-specific Actinomycetales ASVs were not present in seawater samples, though other Actinomycetales ASVs existed in seawater (Supplementary Fig. 8). Pyrosome-specific Actinomycetales ASVs were highly similar (99–100%) to existing sequences from a wide range of sources including sponges, plant hosts, wastewater, and ice (Supplementary Table 4). Actinomycetes produce diverse bioactive secondary metabolites with antibacterial and antifungal properties^75,76^ and provide hosts with chemical defenses,^73,77^ an interesting possibility for pyrosomes. Finally, three ASVs were identified as the cooperative predatory microorganism Myxococcales (Supplementary Table 4).^78,79^ As broad spectrum predators, Myxococcales appear to regulate microbial structure and protect hosts from pathogens and disease^80–83^ through secretion of vesicles^84^ and antimicrobial enzymes.^85^ The presence of Amylibacter, Planctomycetes, Actinomycetes, and Myxococcales suggest that pyrosome colonies are living hosts to a complex surface-associated microbial community with an active network of microbial predator–prey relationships. The known physiologies of these taxa indicate a dynamic balance between biofilm growth, fueled by pyrosome exudates and seawater nutrients, and controlled through the production of antimicrobial compounds and predatory behavior.

### Pyrosomes contain multiple lineages of bioluminescent taxa

Pyrosomes are known for their bioluminescence, however, the bioluminescent mechanism is a subject of debate. A luciferase similar to the cnidarian luciferase RLuc is present in the pyrosome transcriptome, suggesting intrinsic bioluminescence of the pyrosome.^86^ However, several lines of evidence point to microbial symbionts as the agents of pyrosome bioluminescence including: the presence of bioluminescent *Photobacterium,*^87^ high bacterial luciferase activity in luminous organelles,^88^ the propagation of bioluminescence along pyrosome colonies without connective tissue,^89^ variation in the rates of individual zooid responses to light, and increased zooid recruitment with increased luminescence.^20^ As bioluminescence may be the basis for predator evasion and inter-colony communication, identifying the mechanism and potential microbial symbionts that provide bioluminescence is key to understanding pyrosome biology.

We discovered 14 ASVs from bioluminescent genera *Aliivibrio*, *Vibrio*, and *Photobacterium* (Supplementary Table 8). These ASVs were either pyrosome specific or pyrosome enriched (Fig. 4). We hypothesize that pyrosome bioluminescence originates from at least one of these symbionts. Notably, individual pyrosomes had different combinations of ASVs from bioluminescent taxa (Fig. 4A). ASV67, a *Photobacterium* related to known luminescent clades (Fig. 4B), was present in all but one pyrosome. Three other *Photobacterium* ASVs were related to both bioluminescent (Clade I) and non-bioluminescent (Clade II) *Photobacterium*. *Aliivibrio*, represented by ASV557, was present in two pyrosomes and was identical to *A. fischeri* (Supplementary Table 8 and Fig. 4B), the bioluminescent symbiont of the Hawaiian bobtail squid *E. scolopes*. The sporadic distribution of *Aliivibrio* among the pyrosomes does not rule out its involvement in pyrosome bioluminescence. In the symbiosis between *A. fischeri* and *E. scolopes* cultivation of the symbiont reoccurs daily from low concentrations.^90^ *Vibrio* was represented by nine ASVs with varying phylogenetic relationships to known *Vibrio* (Supplementary Table 8 and Fig. 4B). ASV2 and ASV5 had the highest relative abundances and formed a separate group from existing sequences. The high abundance of ASV2 and ASV5 may indicate their concentration in a luminescent organ. Alternatively, *Vibrio* is a diverse group also responsible for pathogenesis^91^ and our short 16S rRNA gene reads limit robust reconstruction of *Vibrio* phylogeny.^47^ These results are consistent with reports of *Photobacterium* within the pyrosome light organ.^87^

The presence of coexisting putative bioluminescent taxa suggests that pyrosome bioluminescence could be the product of multiple microbial taxa, consistent with functional redundancy in animal microbiomes.^92,93^ Other studies of pyrosomes also detected multiple potentially bioluminescent microbial taxa.^87^ Specific to bioluminescence, fish light organs exhibit such functional redundancy with *Photobacterium* cosymbiosis.^94^ However, host and symbiont phylogeny in fish light organs are not congruent, and suggest acquisition of the symbionts from the environment.^95^ Further work on pyrosomes could demonstrate whether the unique bioluminescence of pyrosomes are an emergent property of coexisting luminescent taxa, arise from a single symbiont, and reveal the degree of congruency between symbionts and host evolution.

### Pyrosomes graze diverse microbes with a preference for eukaryotic phytoplankton

Pyrosomes capture planktonic microorganisms by filtering seawater across a mucus mesh.^96,97^ While several studies have documented capture of eukaryotic phytoplankton,^1,4,5,18^ little is understood about the selectivity or efficiency of microbial prey capture by pyrosomes, especially their consumption of abundant marine microbial heterotrophs not detected by pigment-based methods. Given their 0.6 µm mesh pore size,^98^ we expect retention of microbial prey ranging from small Bacteria and Archaea to large eukaryotic microbes. To identify microbial prey, we examined pyrosomes for the presence of well-characterized abundant and free-living marine microorganisms. Specifically, these potential prey included *Pelagibacter (SAR11)*, Marinimicrobia, Euryarchaeota, Thaumarchaeota, *Synechococcus*, and eukaryotic phytoplankton.

**Fig. 5:**
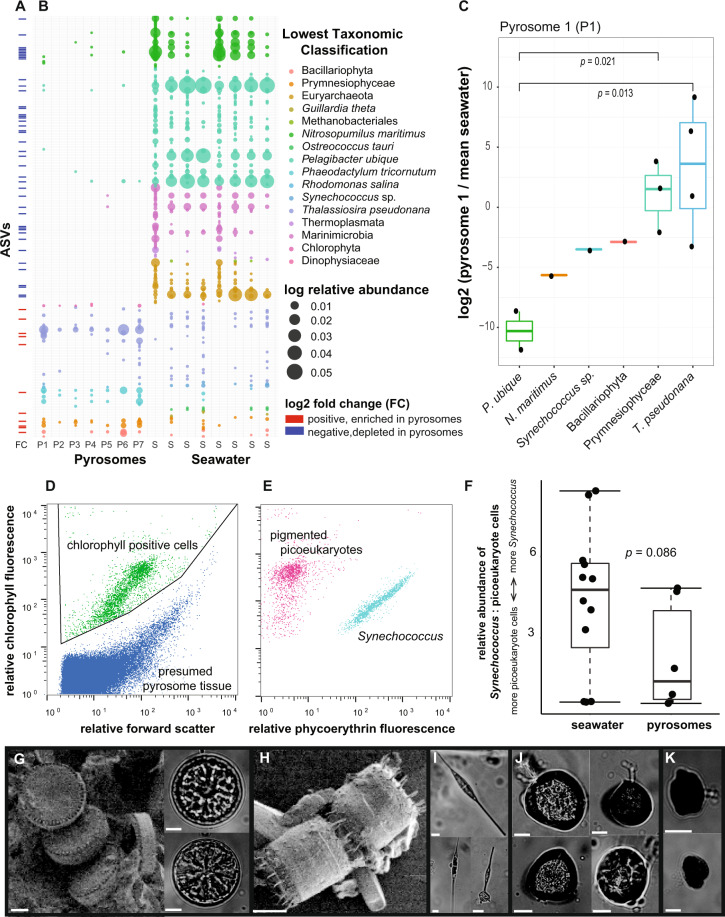
Pyrosomes retain ASVs from multiple free-living marine microbial lineages as prey. **A** Sign of log2 fold change (FC) for ASVs significantly differentially abundant (adjusted *p* value < 0.01) between pyrosomes and seawater. **B** Distribution and relative abundance of free-living potential prey ASVs across pyrosomes and seawater samples colored by lowest level of taxonomic classification. **C** log2 fold change of retained prey ASVs in an individual pyrosome (pyrosome 1) relative to mean relative abundance of each ASV in seawater samples. Comparison of means (*t*-test) indicates that *T. pseudonana* and Prymnesiophytes are significantly more abundant than *P. ubique*. Tests for taxa with only one ASV were not performed. **D** The presence of chlorophyll positive cells in pyrosomes determined by flow cytometry. **E** Chlorophyll positive cells were identified as pigmented picoeukaryotes (PPE) with chlorophyll but no phycoerythrin or *Synechococcus* with chlorophyll and phycoerythrin. **F** The relative abundance of *Synechococcus* to pigmented picoeukaryotes in seawater and pyrosomes was not significantly different (Welch’s *t* test, *p* value = 0.086). Images of phytoplankton found within pyrosomes include: **G** centric diatoms (38*, 47, 43 µm), **H** centric diatoms: *Thalassiosira* sp. (16 µm*), **I** pennate diatoms (84, 120, 44 µm), **J** flagellates (40, 33, 24, 30 µm), and **K** nanozooplankton (13, 17 µm). Sizes denote minimum dimension. Scale bars are 10 µm. Asterisks (*) denote images captured with eSEM; remaining images captured with compound light microscopy.

We found evidence for retention of microbial prey across nearly all tested free-living microbial lineages and predicted cell sizes, but observed enhanced retention of large eukaryotic phytoplankton. Diverse eukaryotic phytoplankton chloroplast ASVs were significantly enriched in pyrosomes relative to seawater (Fig. 5A, B), some of which were in the pyrosome core (Supplementary Fig. 7), and included centric diatoms (*Thalassiosira pseudonana*), pennate diatoms (*Phaeodactylum tricornutum*), and prymnesiophytes. ASVs from cryptophytes, dinoflagellates, and prasinophytes were also present in pyrosomes but were not significantly different from seawater. ASVs from the smaller planktonic microbes *Synechococcus*, *Pelagibacter*, Marinimicrobia, Euryarchaeota, and Thaumarchaeota were very abundant in seawater samples (Fig. 5B) but present more sporadically across pyrosomes. To test whether the sporadic presence of these ASVs could be due to saturation of sequencing by dominant ASVs (above), we looked at the abundance of the potential prey in an individual pyrosome that was not dominated by a single ASV (pyrosome 1) (Fig. 1). Pyrosome 1 had no other characteristics that distinguished it from other pyrosome samples. We found that ASVs from *Synechococcus*, *Pelagibacter*, and *Nitrosopumilus* were present, and that *T. pseudonana* and Prymnesiophyceae were significantly more abundant than *Pelagibacter* (*p* value < 0.05). Single ASVs representing the other taxa precluded statistical tests. The retention of the smallest marine microbes (i.e., *Pelagibacter*) by pyrosomes is consistent with observations in other pelagic tunicates where cells smaller than mesh openings are consumed efficiently due to direct interception on mucus fibers.^99^ The lower retention of *Pelagibacter* in pyrosomes, relative to eukaryotic phytoplankton, is consistent with low retention of *Pelagibacter* in appendicularians and ascidians, due to unique microbial surface membrane properties.^100^ However, we cannot rule out size as a major factor in determining pyrosome retention of microbial prey.

Five of the eukaryotic phytoplankton ASVs were also observed by microscopy and included Bacillariophyta, Prymnesiophyceae, *Thalassiosira sp*., Chlorophyta, and Dinophyceae (Fig. 5G–K). Cells ranged from 1 to 120 µm with the majority of the cells > 10 µm. These results were consistent with the sequence results showing grazing of large phytoplankton. Flow cytometry analysis of pyrosomes also revealed retention of chlorophyll-containing cells including *Synechococcus* and PPE (Fig. 5D), also consistent with sequence results (Fig. 5B). To test whether pyrosomes were selectively feeding on one phytoplankton group, we compared the relative abundance of *Synechococcus* to PPE in the seawater and pyrosomes. The ratio of *Synechococcus* to PPE was 1.9-fold higher in seawater than in pyrosomes, but this difference was not significant (Welch’s *t* test, *p* value = 0.09). Thus, these results do not support positive selection for larger phytoplankton over small cells. However, the robust quantification of phytoplankton relative abundances enabled by flow cytometry did reveal large individual variation in prey content. One caveat here is that the pulverization of pyrosome tissue prior to flow cytometric analysis may have destroyed some eukaryotic cells, leading to underestimation of the number of retained eukaryotes. Possibly, the vertical and horizontal migration of pyrosomes^3^ means coexisting pyrosomes fed on seawater from different depths or locations. Alternatively, differences between individual pyrosomes may reflect colony size, age, or digestion rates as observed in other gelatinous organisms.^101,102^ In addition, 16S rRNA gene sequencing for detection of eukaryotic phytoplankton presents challenges related to phylogenetic resolution.^103^ Also, phytoplankton plastids are present in different locations intracellularly and occur in different numbers across taxa,^104^ leading to different extraction efficiencies and detection via sequencing.

This study is the first to demonstrate that pyrosomes retain a wide range of microbial prey, not only large eukaryotic phytoplankton. We found that pyrosomes preferentially concentrate large eukaryotic phytoplankton relative to smaller picocyanobacteria, Bacteria, and Archaea. Given high pyrosome seawater filtration rates^1,5^ and massive abundances during blooms^13,14,30,31^ reaching up to 95 colonies per m^2^,^2^ these observations suggest a major role for pyrosomes in restructuring marine microbial communities and microbe-driven biogeochemical cycling. In addition to physical parameters, such as temperature and currents, shifting availability of microbial prey may regulate pyrosome blooms. Thus, monitoring or modeling microbial community structure alongside gelatinous zooplankton may serve as a tool for predicting blooms and global contributions to the carbon cycle.^105^ Additional experiments, particularly in situ grazing experiments,^106^ will be required to robustly quantify pyrosome feeding preferences and rates.

## Conclusions

Our results reveal a distinct pyrosome microbiome that is dominated by a few novel taxa, suggesting host-specific relationships and high density of symbionts associated with pyrosomes. Several taxa were related to known bioluminescent microorganisms, suggesting that the unique bioluminescent properties of pyrosomes are due to a microbial symbiont or multiple taxa in cosymbiosis. Pyrosomes harbored sequences from a wide range of free-living microbial taxa, consistent with grazing on a diversity of microbial cells, however, eukaryotic phytoplankton dominated the retained prey species. Numerous microorganisms from taxa that colonize and control surface microbial communities were present, indicating a complex microbial ecosystem on the surface of pyrosomes. Together these results demonstrate that pyrosome biology is intimately linked to marine microorganisms as symbionts, pathogens, and prey. Pyrosome blooms may alter community structure in microbial food webs, influencing biogeochemical cycling and trophic structure in ecosystems.

## Supplementary information

Supplemental Materials

Supplemental Data

Supplemental Tables

## References

[1] Perissinotto R, Mayzaud P, Nichols PD, Labat JP (2007). Grazing by *Pyrosoma atlanticum* (Tunicata, Thaliacea) in the south Indian Ocean. Mar. Ecol. Prog. Ser..

[2] Drits AV, Arashkevich EG, Semenova TN (1992). *Pyrosoma atlanticum* (Tunicata, Thaliacea): grazing impact on phytoplankton standing stock and role in organic carbon flux. J. Plankton Res..

[3] Henschke N (2019). Large vertical migrations of *Pyrosoma atlanticum* play an important role in active carbon transport. J. Geophys. Res. Biogeosci..

[4] Schram, J. B., Sorensen, H. L., Brodeur, R. D., Galloway, A. W. E. & Sutherland, K. R. Abundance, distribution, and feeding ecology of *Pyrosoma atlanticum* in the Northern California Current. *Mar. Ecol. Prog. Ser*. **651**, 97–110 (2020).

[5] O’Loughlin, J. H. et al. Implications of *Pyrosoma atlanticum* range expansion on phytoplankton standing stocks in the Northern California Current. *Prog. Oceanogr*. **188**, 102424 (2020).

[6] Hobson, E. S. & Chess, J. Trophic relations of the blue rockfish, *Sebastes mystinus*, in a coastal upwelling system off northern California. in *Fishery Bulletin*, Vol. **86**, 715–743 (National Marine Fisheries Service, 1988).

[7] Bulman CM, He X, Koslow JA (2002). Trophic ecology of the mid-slope demersal fish community off Southern Tasmania, Australia. Mar. Freshw. Res..

[8] Harbison, G. R. The parasites and predators of Thaliacea. in *The Biology of Pelagic Tunicates* (Oxford University Press, 1998).

[9] James GD, Stahl J-C (2000). Diet of southern Buller’s albatross (*Diomedea bulleri bulleri*) and the importance of fishery discards during chick rearing. N. Z. J. Mar. Freshw. Res..

[10] Hedd A, Gales R (2001). The diet of shy albatrosses (*Thalassarche cauta*) at Albatross Island, Tasmania. J. Zool..

[11] Childerhouse S, Dix B, Gales N (2001). Diet of New Zealand sea lions (*Phocarctos hookeri*) at the Auckland Islands. Wildl. Res..

[12] Lindley JA, Hernández F, Scatllar J, Docoito J (2001). *Funchalia* sp. (Crustacea: Penaeidae) associated with *Pyrosoma atlanticum* (Thaliacea: Pyrosomidae) off the Canary Islands. J. Mar. Biol. Assoc. UK.

[13] Lebrato M, Jones DOB (2009). Mass deposition event of *Pyrosoma atlanticum* carcasses off Ivory Coast (West Africa). Limnol. Oceanogr..

[14] Archer SK (2018). Pyrosome consumption by benthic organisms during blooms in the northeast Pacific and Gulf of Mexico. Ecology.

[15] McFall-Ngai M (2013). Animals in a bacterial world, a new imperative for the life sciences. Proc. Natl Acad. Sci..

[16] Sherr E. & Sherr B. Understanding roles of microbes in marine pelagic food webs: a brief history. in *Microbial Ecology of the Oceans* 27–44 (John Wiley & Sons Ltd, 2008).

[17] Falkowski PG, Fenchel T, Delong EF (2008). The microbial engines that drive earth’s biogeochemical cycles. Science.

[18] Décima M, Stukel MR, López-López L, Landry MR (2019). The unique ecological role of pyrosomes in the Eastern Tropical Pacific. Limnol. Oceanogr..

[19] Gauns M, Mochemadkar S, Pratihary A, Roy R, Naqvi SWA (2015). Biogeochemistry and ecology of *Pyrosoma spinosum* from the Central Arabian Sea. Zool. Stud..

[20] Bowlby MR, Widder EA, Case JF (1990). Patterns of stimulated bioluminescence in two pyrosomes (Tunicata: Pyrosomatidae). Biol. Bull..

[21] Haddock SHD, Moline MA, Case JF (2010). Bioluminescence in the sea. Annu. Rev. Mar. Sci..

[22] Swift, E., Biggley, W. H. & Napora, T. A. The bioluminescence emission spectra of *Pyrosoma atlanticum*, *P. spinosum* (Tunicata), *Euphausia tenera* (Crustacea) and *Gonostoma* sp. (Pisces). *J. Mar. Biol. Assoc. UK* **57**, 817–823 (1977).

[23] Martínez‐García M (2008). Ammonia-oxidizing *Crenarchaeota* and nitrification inside the tissue of a colonial ascidian. Environ. Microbiol..

[24] Donia MS (2011). Complex microbiome underlying secondary and primary metabolism in the tunicate-Prochloron symbiosis. Proc. Natl Acad. Sci..

[25] Kwan JC (2014). Host control of symbiont natural product chemistry in cryptic populations of the tunicate *Lissoclinum patella*. PLoS ONE.

[26] Purcell JE, Arai MN (2001). Interactions of pelagic cnidarians and ctenophores with fish: a review. Hydrobiologia..

[27] Delannoy CMJ, Houghton JDR, Fleming NEC, Ferguson HW (2011). Mauve stingers (*Pelagia noctiluca*) as carriers of the bacterial fish pathogen *Tenacibaculum maritimum*. Aquaculture..

[28] Lee, M. D., Kling, J. D., Araya, R. & Ceh, J. Jellyfish life stages shape associated microbial communities, while a core microbiome is maintained across all. *Front. Microbiol*. **9**, 1534 (2018). 10.3389/fmicb.2018.01534 PMC6052147 30050517

[29] Troussellier, M., Escalas, A., Bouvier, T. & Mouillot, D. Sustaining rare marine microorganisms: macroorganisms as repositories and dispersal agents of microbial diversity. *Front. Microbiol.* **8** (2017). 10.3389/fmicb.2017.00947 PMC5447324 28611749

[30] Brodeur, R. et al. An unusual gelatinous plankton event in the NE Pacific: the Great Pyrosome Bloom of 2017. PICES Press; Sidney Vol. **26**, 22–27 (Winter, 2018).

[31] Sutherland KR, Sorensen HL, Blondheim ON, Brodeur RD, Galloway AWE (2018). Range expansion of tropical pyrosomes in the northeast Pacific Ocean. Ecology.

[32] Miller, R. R. et al. Distribution of pelagic Thaliaceans, *Thetys vagina* and *Pyrosoma Atlanticum*, during a period of mass occurrence within the California current. *CalCOFI Rep.* **60**, (2019).

[33] Guigand, C. M., Cowen, R. K., Llopiz, J. K. & Richardson, D. E. A coupled asymmetrical multiple opening closing net with environmental sampling system. *Mar. Technol. Soc. J*. **39**, 22–24 (2005).

[34] Parada AE, Needham DM, Fuhrman JA (2016). Every base matters: assessing small subunit rRNA primers for marine microbiomes with mock communities, time series and global field samples. Environ. Microbiol..

[35] Callahan BJ (2016). DADA2: high-resolution sample inference from Illumina amplicon data. Nat. Methods.

[36] Cole JR (2014). Ribosomal Database Project: data and tools for high throughput rRNA analysis. Nucleic Acids Res..

[37] Wang Q, Garrity GM, Tiedje JM, Cole JR (2007). Naïve Bayesian classifier for rapid assignment of rRNA sequences into the new bacterial taxonomy. Appl. Environ. Microbiol..

[38] O’Leary NA (2016). Reference sequence (RefSeq) database at NCBI: current status, taxonomic expansion, and functional annotation. Nucleic Acids Res..

[39] Johnson M (2008). NCBI BLAST: a better web interface. Nucleic Acids Res..

[40] McMurdie PJ, Holmes S (2013). phyloseq: an R package for reproducible interactive analysis and graphics of microbiome census data. PLoS ONE.

[41] Clarke KR (1993). Non-parametric multivariate analyses of changes in community structure. Aust. J. Ecol..

[42] Love MI, Huber W, Anders S (2014). Moderated estimation of fold change and dispersion for RNA-seq data with DESeq2. Genome Biol..

[43] McMurdie PJ, Holmes S (2014). Waste not, want not: why rarefying microbiome data is inadmissible. PLoS Comput. Biol..

[44] Duperron, S. *Microbial Symbioses* 168 p. (Elsevier, 2016).

[45] Schmitt S (2012). Assessing the complex sponge microbiota: core, variable and species-specific bacterial communities in marine sponges. ISME J..

[46] Gloor, G. B., Macklaim, J. M., Pawlowsky-Glahn, V. & Egozcue, J. J. Microbiome datasets are compositional: and this is not optional. *Front. Microbiol.* **8** (2017). 10.3389/fmicb.2017.02224 PMC5695134 29187837

[47] Urbanczyk H, Ast JC, Higgins MJ, Carson J, Dunlap PV (2007). Reclassification of *Vibrio fischeri, Vibrio logei, Vibrio salmonicida* and *Vibrio wodanis* as *Aliivibrio fischeri* gen. nov., comb. nov., *Aliivibrio logei* comb. nov., *Aliivibrio salmonicida* comb. nov. and *Aliivibrio wodanis* comb. nov. Int. J. Syst. Evol. Microbiol..

[48] Stecher G, Tamura K, Kumar S (2020). Molecular Evolutionary Genetics Analysis (MEGA) for macOS. Mol. Biol. Evol..

[49] Letunic I, Bork P (2019). Interactive Tree Of Life (iTOL) v4: recent updates and new developments. Nucleic Acids Res..

[50] Booth BC (1994). Marine phytoplankton. A guide to naked flagellates and coccolithophorids (C. R. Tomas [ed.]). Limnol. Oceanogr..

[51] Halse, G. R. & Syvertsen, E. E. Chapter 2—marine diatoms. in *Identifying Marine Diatoms and Dinoflagellates* (ed. Tomas C. R.) 5–385 (Academic Press, 1996).

[52] Steidinger, K. A. & Tangen, K. Chapter 3—dinoflagellates. in *Identifying Marine Diatoms and Dinoflagellates* (ed. Tomas C. R.) 387–584 (Academic Press, 1996).

[53] Daniels C, Breitbart M (2012). Bacterial communities associated with the ctenophores *Mnemiopsis leidyi* and *Beroe ovata*. FEMS Microbiol. Ecol..

[54] Kramar MK, Tinta T, Lučić D, Malej A, Turk V (2019). Bacteria associated with moon jellyfish during bloom and post-bloom periods in the Gulf of Trieste (northern Adriatic). PLoS ONE.

[55] Hernandez-Agreda, A., Leggat, W., Bongaerts, P., Herrera, C. & Ainsworth, T. D. Rethinking the coral microbiome: simplicity exists within a diverse microbial biosphere. *mBio* **9**, e00812–18 (2018). 10.1128/mBio.00812-18 PMC6178627 30301849

[56] Webster NS, Bourne D (2007). Bacterial community structure associated with the Antarctic soft coral, *Alcyonium antarcticum*. FEMS Microbiol. Ecol..

[57] Rodrigues CF, Hilário A, Cunha MR, Weightman AJ, Webster G (2011). Microbial diversity in Frenulata (Siboglinidae, Polychaeta) species from mud volcanoes in the Gulf of Cadiz (NE Atlantic). Antonie Van Leeuwenhoek.

[58] McCann J, Stabb EV, Millikan DS, Ruby EG (2003). Population dynamics of *Vibrio fischeri* during Infection of *Euprymna scolopes*. Appl. Environ. Microbiol..

[59] Hammann S, Moss A, Zimmer M (2015). Sterile surfaces of *Mnemiopsis leidyi*; (Ctenophora) in bacterial suspension—a key to invasion success?. Open J. Mar. Sci..

[60] Hammer, T. J., Sanders, J. G. & Fierer, N. Not all animals need a microbiome. *FEMS Microbiol. Lett*. **366**, fnz117 10.1093/femsle/fnz117 (2019). 10.1093/femsle/fnz117 31132110

[61] Nedashkovskaya OI, Kukhlevskiy AD, Zhukova NV, Kim SB (2016). *Amylibacter ulvae* sp. nov., a new alphaproteobacterium isolated from the Pacific green alga *Ulva fenestrata*. Arch. Microbiol..

[62] Burke C, Thomas T, Lewis M, Steinberg P, Kjelleberg S (2011). Composition, uniqueness and variability of the epiphytic bacterial community of the green alga Ulva australis. ISME J..

[63] Catão, E. C. P. et al. Shear stress as a major driver of marine biofilm communities in the NW Mediterranean Sea. *Front. Microbiol*. **10** (2019). 10.3389/fmicb.2019.01768 PMC6774042 31608016

[64] Chafee M (2018). Recurrent patterns of microdiversity in a temperate coastal marine environment. ISME J..

[65] Bondoso J (2015). *Roseimaritima ulvae* gen. nov., sp. nov. and *Rubripirellula obstinata* gen. nov., sp. nov. two novel planctomycetes isolated from the epiphytic community of macroalgae. Syst. Appl. Microbiol..

[66] Zhu P, Li Q, Wang G (2008). Unique microbial signatures of the Alien Hawaiian marine sponge *Suberites* zeteki. Microb. Ecol..

[67] Pimentel-Elardo S, Wehrl M, Friedrich AB, Jensen PR, Hentschel U (2003). Isolation of planctomycetes from *Aplysina* sponges. Aquat. Microb. Ecol..

[68] da Silva Oliveira FA (2013). Microbial epibionts of the colonial ascidians *Didemnum galacteum* and *Cystodytes* sp. Symbiosis.

[69] Yakimov MM (2006). Phylogenetic survey of metabolically active microbial communities associated with the deep-sea coral *Lophelia pertusa* from the Apulian plateau, Central Mediterranean Sea. Deep Sea Res. A Oceanogr. Res. Pap..

[70] Duque-Alarcón A, Santiago-Vázquez LZ, Kerr RG (2012). A microbial community analysis of the octocoral *Eunicea fusca*. Electron. J. Biotechnol..

[71] Wiegand S, Jogler M, Jogler C (2018). On the maverick Planctomycetes. FEMS Microbiol. Rev..

[72] Lage, O. M. & Bondoso, J. Planctomycetes and macroalgae, a striking association. *Front. Microbiol*. **5** (2014). 10.3389/fmicb.2014.00267 PMC4042473 24917860

[73] Ward AC, Bora N (2006). Diversity and biogeography of marine Actinobacteria. Curr. Opin. Microbiol..

[74] Hahn MW (2009). Description of seven candidate species affiliated with the phylum Actinobacteria, representing planktonic freshwater bacteria. Int. J. Syst. Evol. Microbiol..

[75] Gandhimathi R (2008). Antimicrobial potential of sponge associated marine actinomycetes. J. Mycol. Méd..

[76] Abdelmohsen UR, Bayer K, Hentschel U (2014). Diversity, abundance and natural products of marine sponge-associated actinomycetes. Nat. Prod. Rep..

[77] Wu Z (2005). A new tetrodotoxin-producing actinomycete, *Nocardiopsis dassonvillei*, isolated from the ovaries of puffer fish Fugu rubripes. Toxicon..

[78] Reichenbach H (1999). The ecology of the myxobacteria. Environ. Microbiol..

[79] Marshall RC, Whitworth DE (2019). Is “Wolf-Pack” predation by antimicrobial bacteria cooperative? Cell behaviour and predatory mechanisms indicate profound selfishness, even when working alongside Kin. BioEssays.

[80] Welsh RM (2016). Bacterial predation in a marine host-associated microbiome. ISME J..

[81] Wang Z, Kadouri DE, Wu M (2011). Genomic insights into an obligate epibiotic bacterial predator: *Micavibrio aeruginosavorus* ARL-13. BMC Genomics.

[82] Garcia GD (2013). Metagenomic analysis of healthy and white plague-affected *Mussismilia braziliensis* corals. Microb. Ecol..

[83] Rosales SM (2019). Microbiome differences in disease-resistant vs. susceptible *Acropora* corals subjected to disease challenge assays. Sci. Rep..

[84] Evans AGL (2012). Predatory activity of *Myxococcus xanthus* outer-membrane vesicles and properties of their hydrolase cargo. Microbiology.

[85] Sudo S, Dworkin M (1972). Bacteriolytic enzymes produced by *Myxococcus xanthus*. J. Bacteriol..

[86] Tessler M (2020). A putative chordate luciferase from a cosmopolitan tunicate indicates convergent bioluminescence evolution across phyla. Sci. Rep..

[87] Berger, A. et al. Microscopic and Genetic Characterization of Bacterial Symbionts With Bioluminescent Potential in Pyrosoma Atlanticum. *Frontiers in Marine Science*. **8**10.3389/fmars.2021.606818 (2021).

[88] Leisman G, Cohn DH, Nealson KH (1980). Bacterial origin of luminescence in marine animals. Science.

[89] Mackie GO, Bone Q (1978). Luminescence and associated effector activity in *Pyrosoma* (Tunicata: Pyrosomida). Proc. R. Soc. Lond. B Biol. Sci..

[90] Nyholm SV, McFall-Ngai M (2004). The winnowing: establishing the squid–vibrio symbiosis. Nat. Rev. Microbiol..

[91] Takemura, A. F., Chien, D. M. & Polz M. F. Associations and dynamics of Vibrionaceae in the environment, from the genus to the population level. *Front. Microbiol*. **5** (2014). 10.3389/fmicb.2014.00038 PMC3920100 24575082

[92] Barnes, E. M., Carter, E. L. & Lewis, J. D. Predicting microbiome function across space is confounded by strain-level differences and functional redundancy across taxa. *Front. Microbiol.* **11** (2020). 10.3389/fmicb.2020.00101 PMC7018939 32117131

[93] Tian, L. et al. Deciphering functional redundancy in the human microbiome. *bioRxiv* 176313 10.1101/176313 (2017). 10.1038/s41467-020-19940-1 PMC7719190 33277504

[94] Kaeding AJ (2007). Phylogenetic diversity and cosymbiosis in the bioluminescent symbioses of “*Photobacterium mandapamensis*”. Appl. Environ. Microbiol..

[95] Baker, L. J. et al. Diverse deep-sea anglerfishes share a genetically reduced luminous symbiont that is acquired from the environment. *eLife* **8** e47606 (2019). 10.7554/eLife.47606 PMC6773444 31571583

[96] Godeaux, J. E. A., Bone, Q. & Braconnot, J. C. Anatomy of Thaliacea. in *The Biology of Pelagic Tunicates* (Oxford University Press, 1998).

[97] Alldredge AL, Madin LP (1982). Pelagic tunicates: unique herbivores in the marine plankton. BioScience..

[98] Bone Q, Carre C, Ryan KP (2000). The endostyle and the feeding filter in salps (Tunicata). J. Mar. Biol. Assoc. UK.

[99] Sutherland KR, Madin LP, Stocker R (2010). Filtration of submicrometer particles by pelagic tunicates. Proc. Natl Acad. Sci..

[100] Dadon-Pilosof A (2017). Surface properties of SAR11 bacteria facilitate grazing avoidance. Nat. Microbiol..

[101] Larson RJ (1987). Daily ration and predation by medusae and ctenophores in Saanich Inlet, B.C., Canada. Neth. J. Sea Res..

[102] Suchman CL, Daly EA, Keister JE, Peterson WT, Brodeur RD (2008). Feeding patterns and predation potential of scyphomedusae in a highly productive upwelling region. Mar. Ecol. Prog. Ser..

[103] Bennke CM (2018). The distribution of phytoplankton in the Baltic Sea assessed by a prokaryotic 16S rRNA gene primer system. J. Plankton Res..

[104] Green BR (2011). Chloroplast genomes of photosynthetic eukaryotes. Plant J..

[105] Luo, J. Y. et al. Gelatinous zooplankton-mediated carbon flows in the global oceans: a data-driven modeling study. *Glob. Biogeochem. Cycles*. **34**, e2020GB006704 (2020).

[106] Dadon‐Pilosof A, Lombard F, Genin A, Sutherland KR, Yahel G (2019). Prey taxonomy rather than size determines salp diets. Limnol. Oceanogr..

[107] Brand A, Liz A, Micah A, Marjorie H, Jo S (2015). Beyond Authorship: Attribution, Contribution, Collaboration, and Credit. Learned Publishing..

