# Supplementary material for: Host-specific symbioses and the microbial prey of a pelagic tunicate (Pyrosoma atlanticum)
Source: ISME Commun. 2021 Apr 14;1:11. doi: 10.1038/s43705-021-00007-1 (PMC9723572; doi:10.1038/s43705-021-00007-1)

Supplemental Table 1. Taxonomic assignment of ASVs.

Supplemental Table 2. Raw sequence counts for each sample and each ASV.

Supplemental Table 3. Normalized sequence counts for each sample and each ASV.

Supplemental Table 4. BLAST results and taxonomy for pyrosome specific ASVs.

Supplemental Table 5. BLAST results and taxonomy for pyrosome and seawater shared ASVs.

Supplemental Table 6. BLAST results and taxonomy for pyrosome core ASVs.

Supplemental Table 7. BLAST results and taxonomy for seawater specific ASVs.

Supplemental Table 8. BLAST results and taxonomy for ASVs from potentially-bioluminescent microorganisms.

Supplemental Figure 1. Water column structure at the two stations with temperature (blue) and chlorophyll concentration (green) over depth. Horizontal black lines indicate depths of discrete seawater sampling via CTD rosette. Orange boxes indicate the depth ranges where pyrosomes were collected using the MOCNESS.

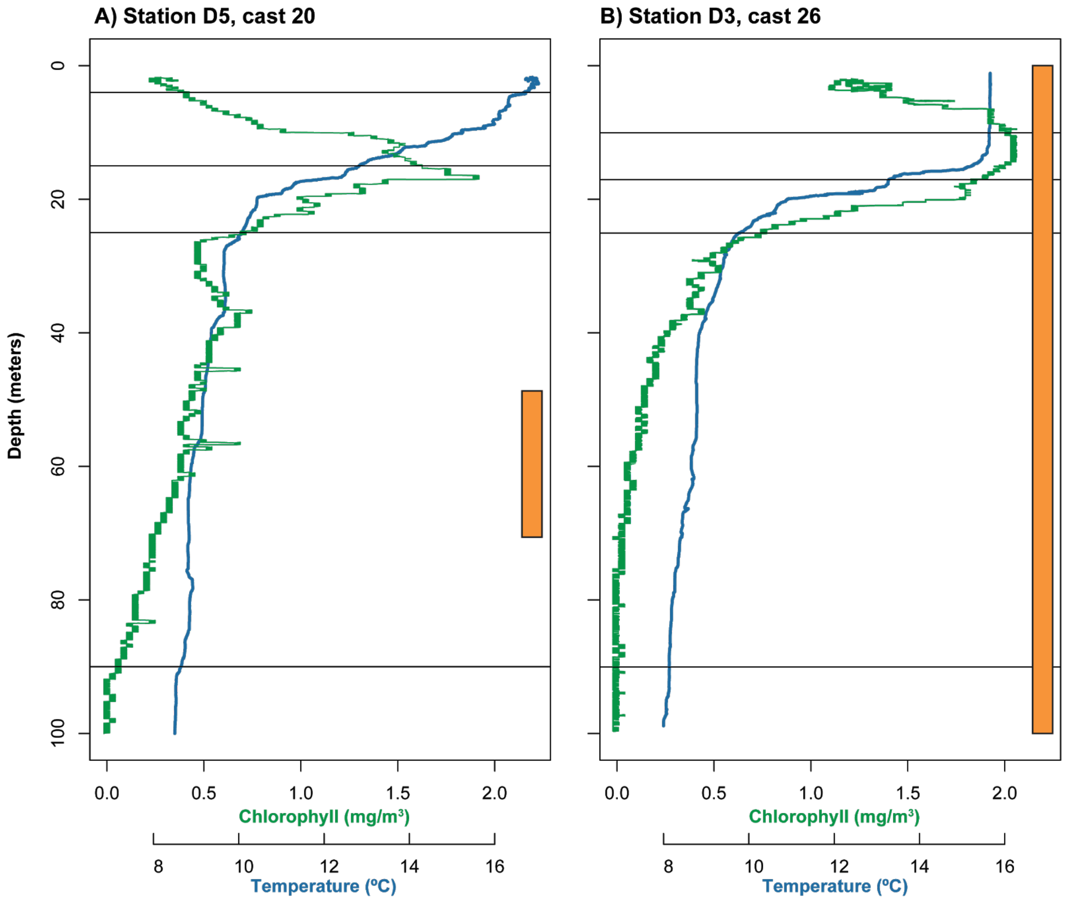

Supplemental Figure 2. A) Number of high quality sequence reads passing filters for each pyrosome ("P") and seawater ("S") sample. B) Percent of standardized sequence read counts belonging to each Phylum from all combined pyrosome and seawater samples.

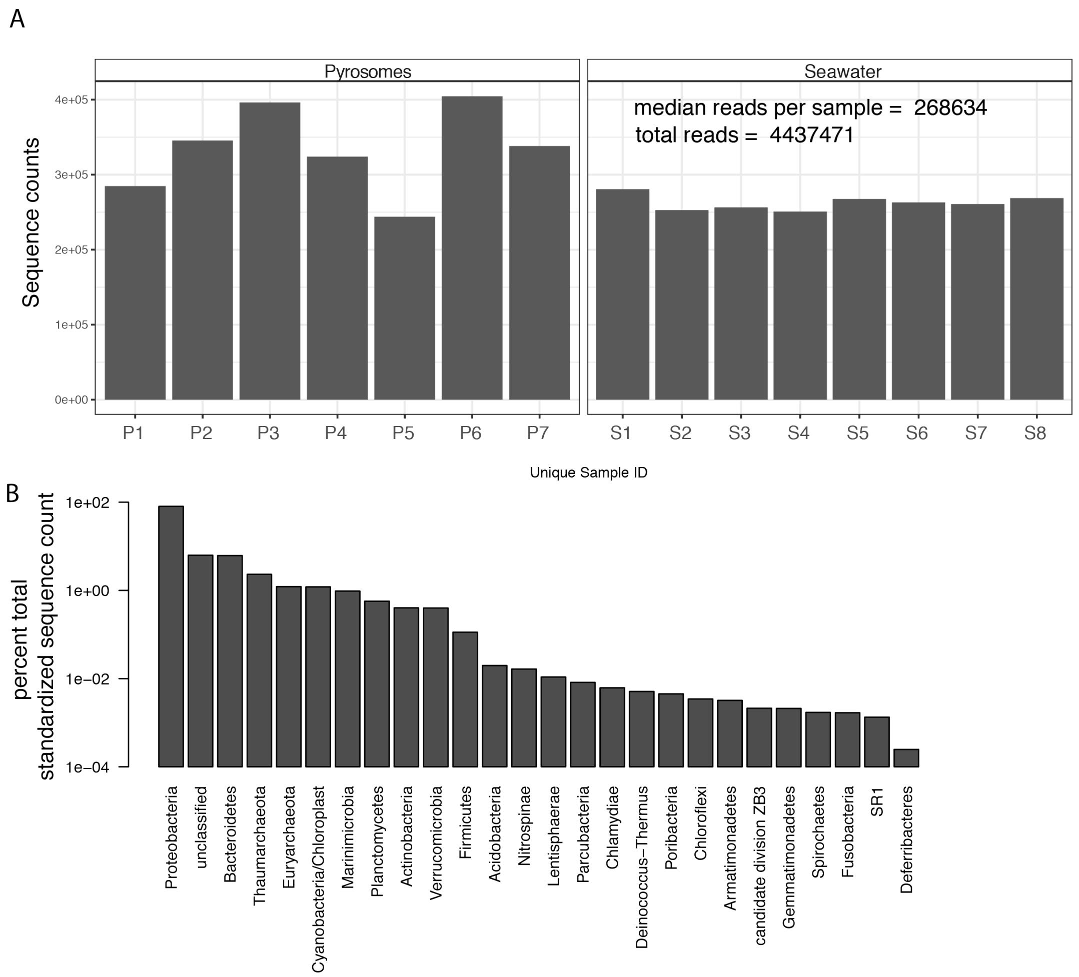

Supplemental Figure 3. Rarefaction curves of seawater samples compared to pyrosome samples with line labels indicating the unique sample identifier. Sample size is the number of sequence reads sampled. Species is the number of ASVs present.

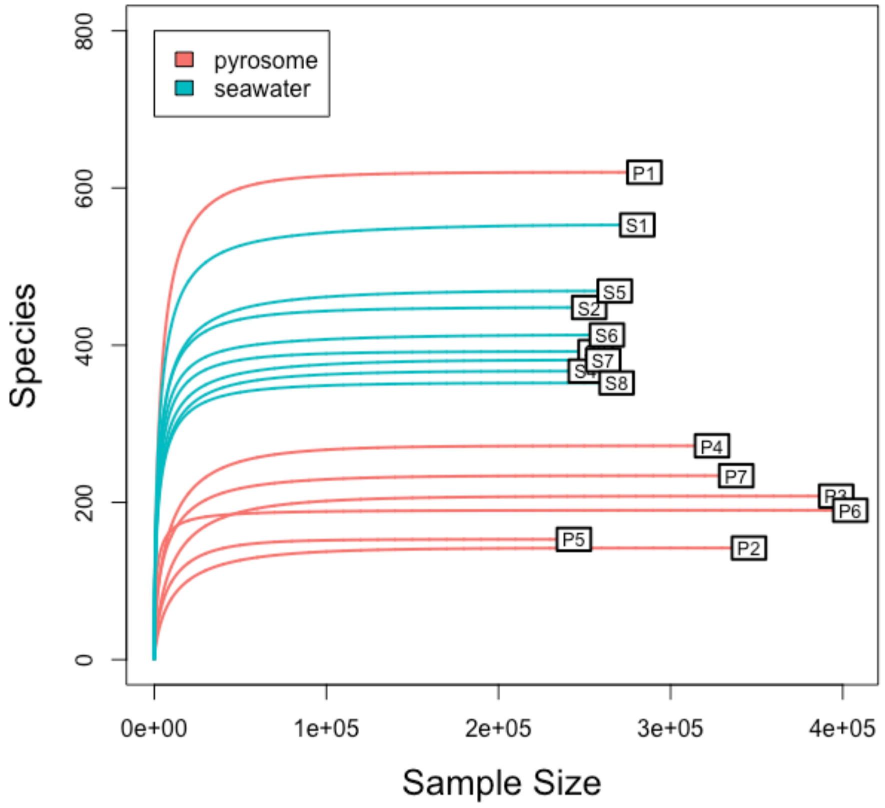

Supplemental Figure 4. NMDS of seawater (A, B) and pyrosome (C, D) Bray Curtis dissimilarities with ANOSIM strength (R statistic) and significance (*p-value*) displayed for groupings by sampling station or depth. In C and D, "n = 2" indicates two overlapping pyrosome samples.

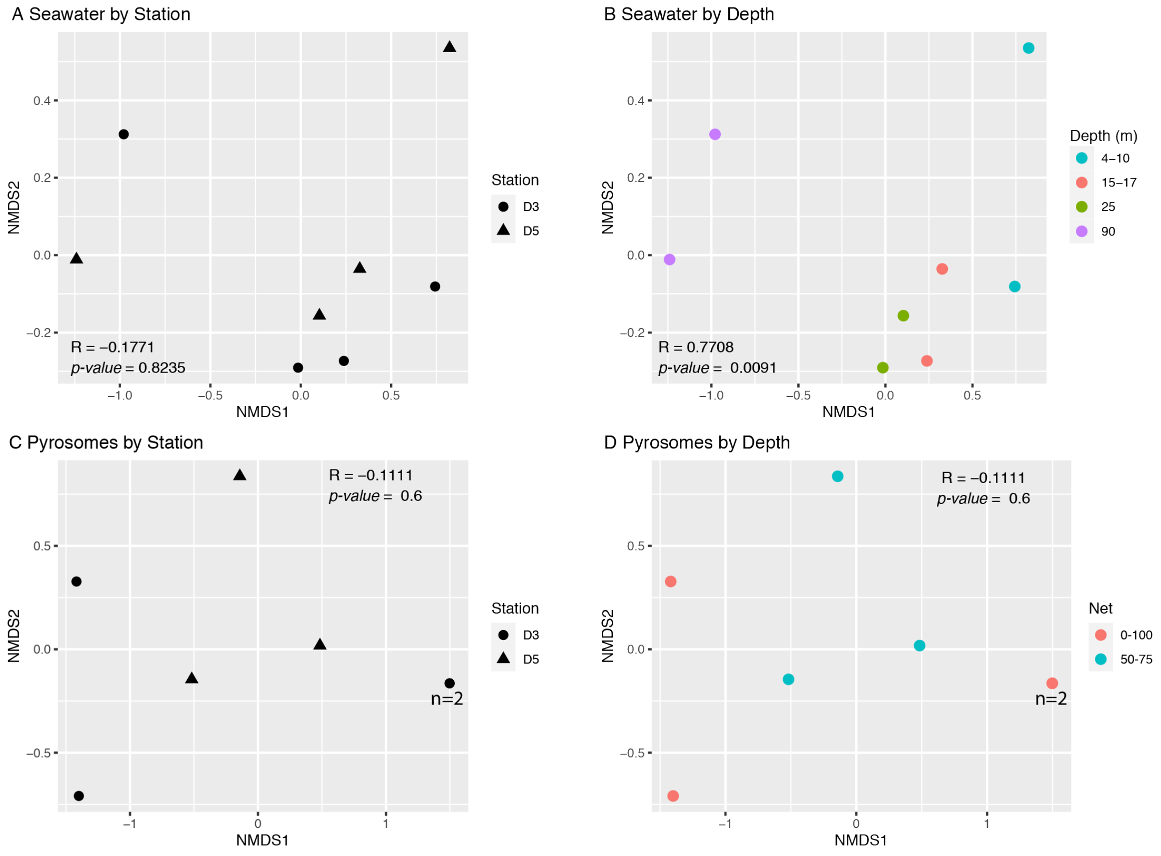

Supplemental Figure 5. Log2 fold change of ASVs found significantly enriched in pyrosomes (positive values, 143 ASVs) or seawater (negative values, 160 ASVs) via *DESeq*2. ASVs are colored and organized by phylum. NA indicates no phylum level classification assigned.

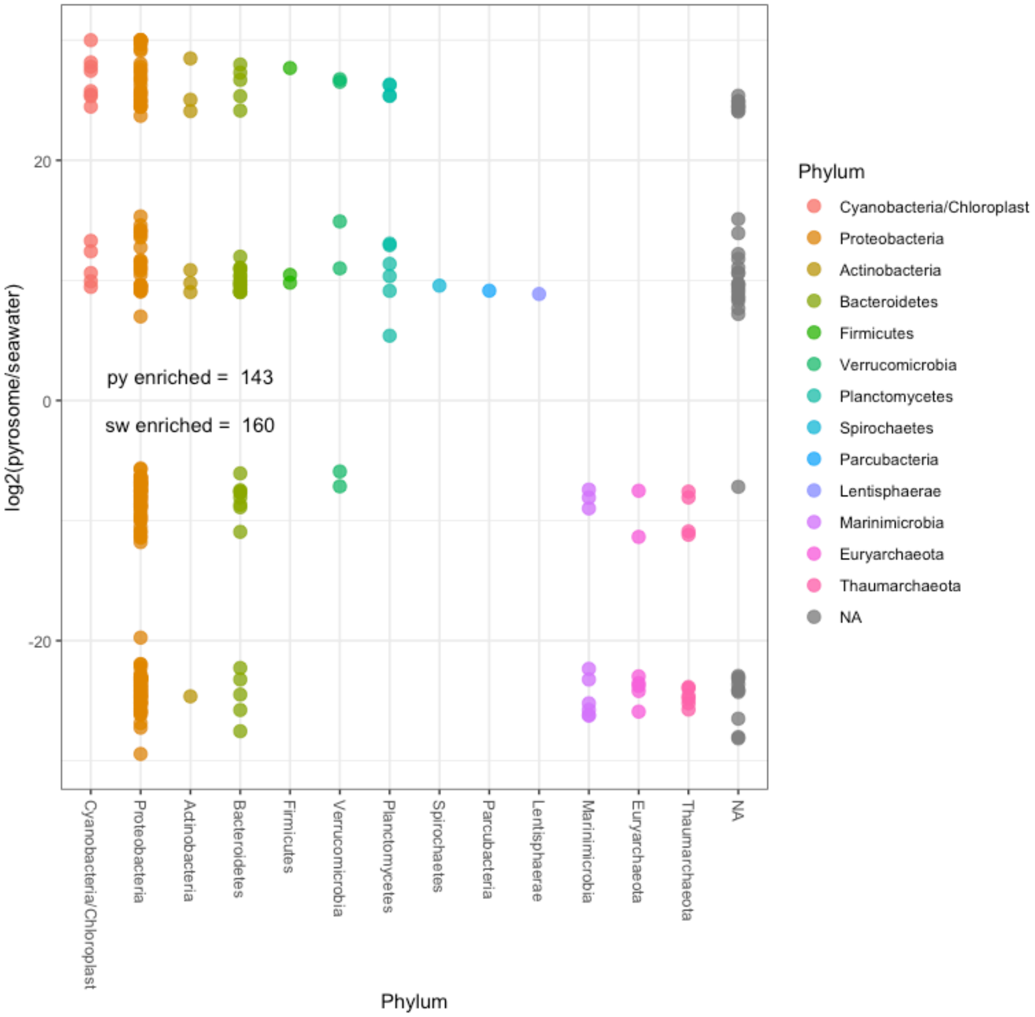

Supplemental Figure 6. Categories or sample sources of top best BLASTn hits of ASVs from pyrosome-specific, seawater-specific, and shared.

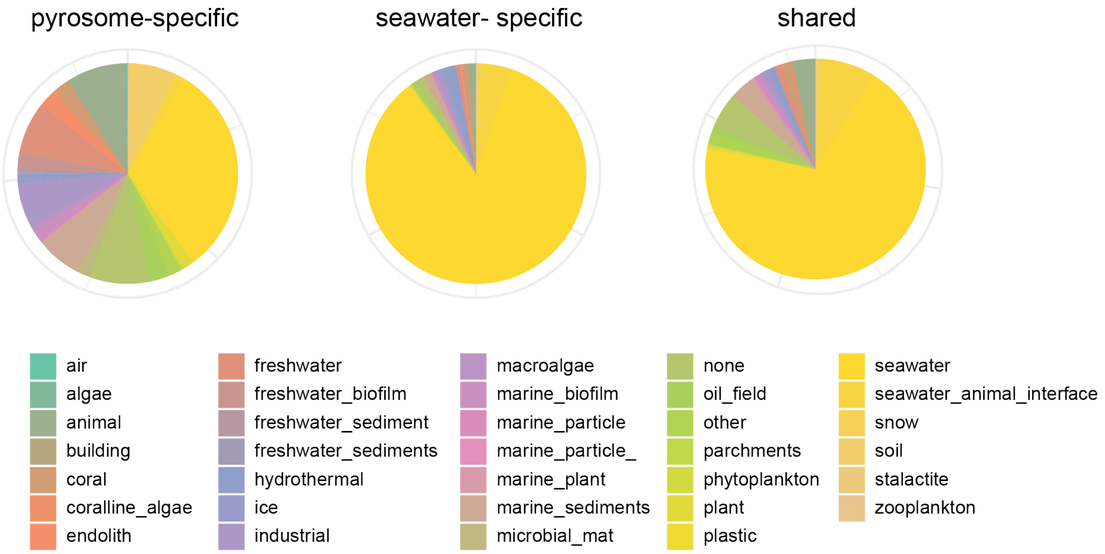

Supplemental Figure 7. Mean relative percent abundance of pyrosome core ASVs across all pyrosome samples.

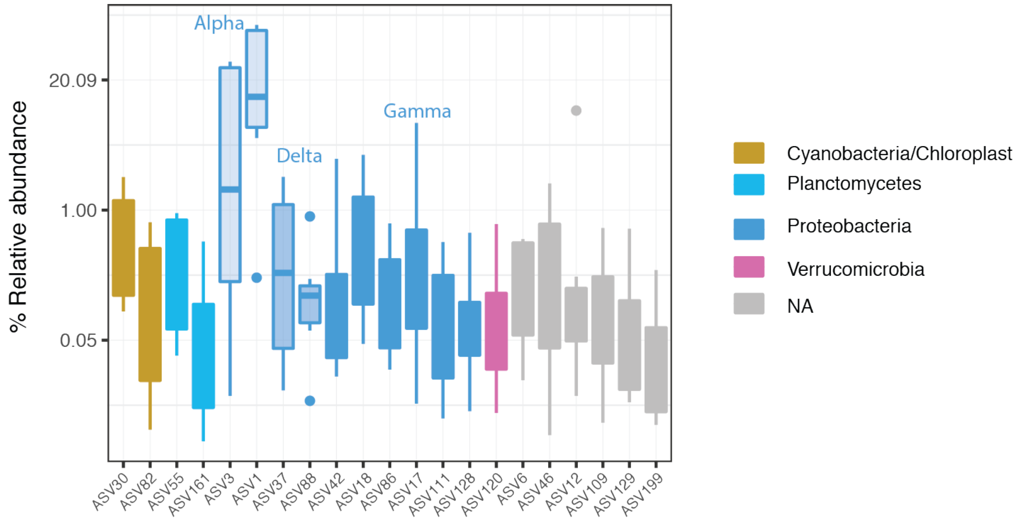

Supplemental Figure 8. Distribution and relative abundance of surface-associated taxa in pyrosomes (P) and seawater (S).

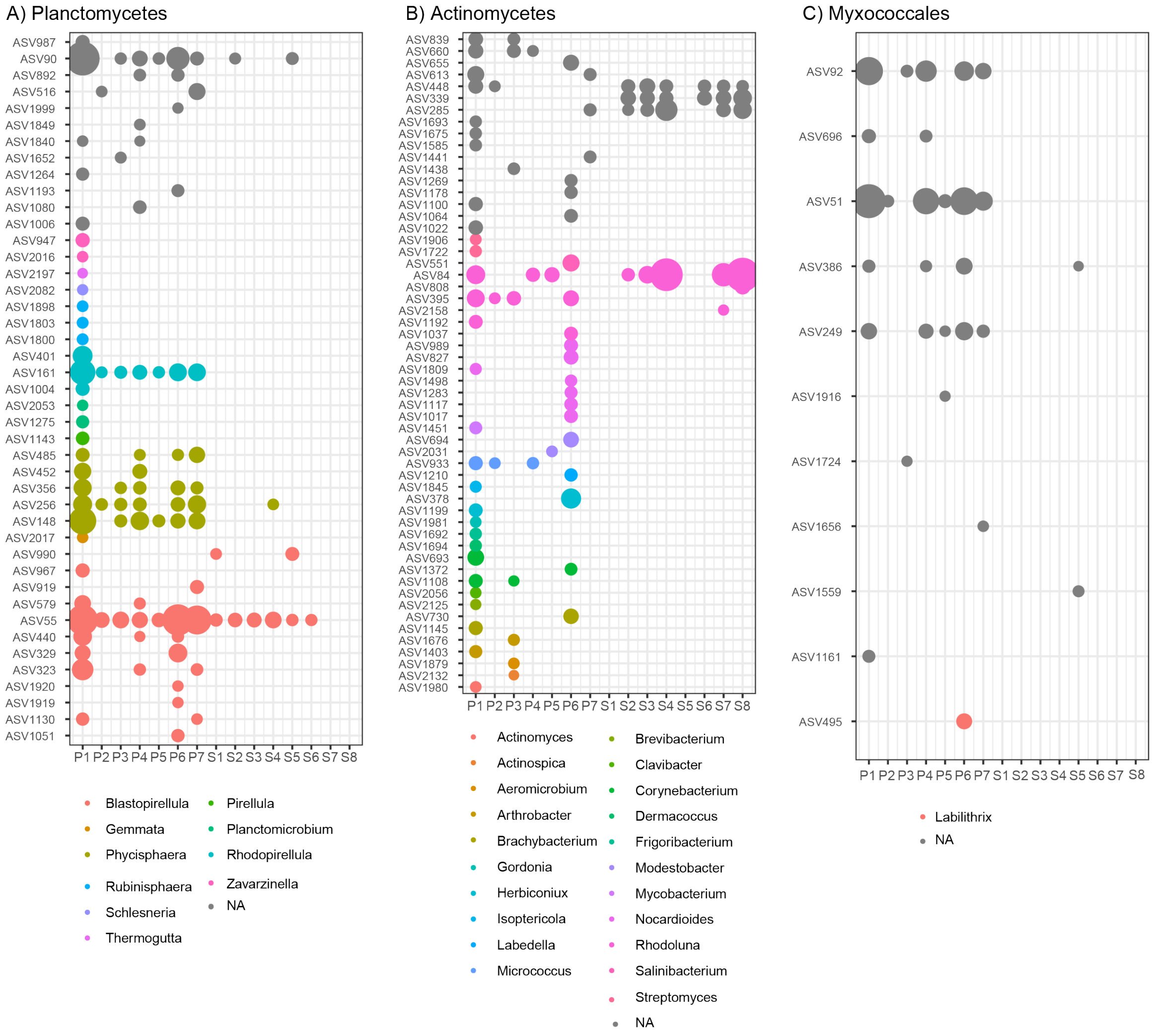

Supplement: Supplementary file 1 — Supplemental Materials [file 43705_2021_7_MOESM1_ESM.pdf]
